# Regulation of dopaminergic function: an [^18^F]-DOPA PET apomorphine challenge study in humans.

**DOI:** 10.1038/tp.2016.270

**Published:** 2017-02-07

**Authors:** S Jauhar, M Veronese, M Rogdaki, M Bloomfield, S Natesan, F Turkheimer, S Kapur, O D Howes

**Affiliations:** 1 Department of Psychosis Studies, Institute of Psychiatry, Psychology and Neuroscience, King’s College, London, UK; 2 Centre for Neuroimaging Sciences, Institute of Psychiatry, Psychology and Neuroscience, King’s College, London, UK; 3 MRC London Institute of Medical Sciences, London, UK; 4 Institute of Clinical Sciences, Department of Medicine, Imperial College London, London, UK

## Abstract

Dopaminergic function has a key role in normal brain function, dopaminergic dysfunction being implicated in numerous neuropsychiatric disorders. Animal studies show that dopaminergic stimulation regulates dopaminergic function, but it is not known whether this exists in humans. In the first study (study 1), we measured dopamine synthesis capacity (indexed as *K_i_*^cer^) to identify the relationship between baseline and change in *K_i_*^cer^ under resting conditions for comparison with effects of dopaminergic stimulation. In the second study (study 2), we used a within-subjects design to test effects of dopaminergic stimulation on dopamine synthesis capacity. In study 1, eight volunteers received two ^18^F-DOPA scans on separate days, both at rest. In study 2, 12 healthy male volunteers received two ^18^F-DOPA positron emission tomographic (PET) scans after treatment with either the dopamine partial agonist apomorphine (0.03 or 0.005 mg kg^−1^) or placebo. In study 1, no significant correlation was found between baseline and change in dopamine synthesis capacity between scans (*r*=−0.57, *n*=8, *P*=0.17, two-tailed). In study 2, a significant negative correlation was found between baseline dopamine synthesis capacity and percentage change in dopamine synthesis capacity after apomorphine challenge (*r*=−0.71, *n*=12, *P*=0.01, two-tailed). This correlation was significantly different (*P*<0.01) from the correlation between baseline and change in dopamine synthesis capacity under unstimulated conditions. One-way repeated-measures analysis of variance showed a significant group (study 1/study 2) × time interaction (F(1,18)=11.5, *P*=0.003). Our findings suggest that regulation of dopamine synthesis capacity by apomorphine depends on baseline dopamine function, consistent with dopamine stimulation stabilizing dopaminergic function. Loss of this autoregulation may contribute to dopaminergic dysfunction in brain disorders such as schizophrenia, substance dependence, and Parkinson's disease.

## Introduction

Since its discovery in 1957,^1^ the neuromodulator dopamine has been shown to have a key role in regulating affect, attention, motivation,^2^ reward,^3^ sleep^4^ and voluntary movement.^5^ Variation in dopamine synthesis capacity (DSC) has been linked to alterations in a number of these functions,^6, 7^ and may have a pathoetiological role in a number of neuropsychiatric disorders, including attention deficit hyperactivity disorder (ADHD),^8^ Parkinson’s disease,^9^ schizophrenia^10, 11^ and substance dependence.^12^ Specifically, elevated DSC, as measured by [^18^F]-DOPA, is seen in people with schizophrenia^13, 14^ and in the prodrome to psychosis,^15, 16^ whereas DSC is reduced in ADHD^17^ and Parkinson’s disease.^18^ People with substance dependence also show alterations in DSC in some although not all studies.^19, 20, 21^

Thus, determining the regulation of DSC is likely to be important for understanding and treating a number of brain disorders. Preclinical studies have shown that dopamine agonists at dopamine D_1_ and dopamine D_2_ receptors exert regulatory effects on DSC through three main mechanisms:

1. Terminal negative feedback, through presynaptic autoreceptors (mainly through D_2_ receptors), on dopamine nerve terminals in the striatum.^22, 23, 24^
2. ‘Long loop’ negative feedback, involving a direct striatonigral pathway, stimulated by postsynaptic receptors and an indirect striatopallidal pathway, stimulated by postsynaptic D_2_ receptors.^25, 26^
3. Somatodendritic mechanisms, with dopamine acting on D_2_ receptors on dopamine cell bodies in the midbrain to decrease dopamine neural activity and release.^26, 27^

Apomorphine is considered a potent D_1_ and D_2_ receptor partial agonist (with higher selectivity for D_2_), and is widely used for Parkinson’s disease.^28^ Preclinical studies suggest it is a partial dopamine agonist with 79% and 53% efficacy relative to dopamine at D_2_ receptor D_2__-short_ and D_2-long_ splice variants, respectively.^29^ Besides acting on postsynaptic dopamine receptors, apomorphine has agonist effects on presynaptic autoreceptors,^30, 31^ has been used to measure the distribution of dopamine autoreceptors^32^ and has been shown to have effects on the basal firing rate of dopamine neurons,^33^ and differential effects on locomotor activity in rodents, depending on dosage.^34^

Apomorphine is therefore thought to act via one or more of the autoregulatory mechanisms above, to regulate dopamine function. Supporting this, a study in rhesus monkeys showed that apomorphine reduces DSC, and that this effect was greater in animals with greater baseline DSC.^35^ To date, *in vivo* studies in humans have been limited to people with Parkinson’s disease (PD), one [^11^C]-raclopride study finding evidence of reduced dopamine release following amphetamine,^36^ and a study in early and late PD, finding reduced DSC in early PD, but the numerically opposite effect in late PD.^37^ The study of apomorphine in Parkinson’s disease is problematic when looking at function in non-disease populations, as animal literature suggests autoreceptor sensitivity to be affected when damage to the nigrostriatal system has occurred.^38^

Therefore, although these findings are consistent with an autoregulatory effect of dopamine agonism in disease, the regulation of DSC in healthy humans remains to be determined. In view of this, we aimed to determine the relationship between baseline dopamine synthesis capacity and the effect of dopamine stimulation, using apomorphine, on dopamine synthesis capacity, indexed using ^18^F-DOPA PET, in healthy humans. In study 1, we determined the reliability of ^18^F-DOPA imaging and the relationship between baseline DSC and change on re-scanning in untreated healthy volunteers to provide reference data for comparison. In study 2, we determined the effect of apomorphine on DSC using doses designed to preferentially act on presynaptic autoreceptors^36, 39^ to test the hypothesis that apomorphine would alter DSC, and that this would depend on baseline DSC.

## Materials and methods

### Ethical approval

Study 1 was approved by the South London and Maudsley/Institute of Psychiatry NHS Trust. Study 2 was approved by the Hammersmith Research Ethics Committee. The Administration of Radioactive Substances Advisory Committee (ARSAC) granted permission to administer [^18^F]-DOPA for both the studies.

### Participants

All the participants gave written informed consent. The participants were recruited through the local media. Inclusion criteria for all the subjects were: male gender, age 18–35, no history of major medical illness, and capacity to give written informed consent. The exclusion criteria for all the participants were: presence of any significant current medical disorder or treatment including history of head injury resulting in loss of consciousness and any neurological disorder; diagnosis of past or current psychiatric disorders using the Structured Clinical Interview for DSM-IV^40^ including alcohol or any other substance dependence or abuse. All the participants provided urine samples on the day of the PET scans to screen for drug use (Monitect HC12, Branan Medical, Irvine, CA, USA), and were excluded if they were positive. No subject was taking psychotropic medication at the time of study participation.

### Study 1: test–retest study to determine the relationship between baseline dopamine synthesis capacity and change in dopamine synthesis capacity under resting (unstimulated) conditions

Eight healthy adults (mean age 23.6±3.5 years, range 19–28 years, five males, six right-handed) participated in this study as part of an ongoing research project.^41^

#### PET data acquisition

Each subject received two ^18^F-DOPA PET scans, administered approximately 2 years apart (mean±SD=113.6±16 weeks). PET imaging was performed on an ECAT/EXACT3D:Siemens/CTI (Knoxville, TN, USA) PET tomograph (spatial resolution: 4.8 (0.2) mm; sensitivity: 69 cps Bq^−1^ ml^−1^). High-resolution images of the whole brain were reconstructed from 95 planes with a section spacing of 2.425 mm. All the participants were asked not to eat or drink (except water), and refrain from alcohol for 12 h before the scan. The subjects received carbidopa (150 mg) and entacapone (400 mg) orally about 1 h before imaging.^42^ The administration of carbidopa and entacapone reduces the formation of radiolabeled ^18^F-DOPA metabolites,^43^ increasing the signal-to-noise ratio.^42^ The 400 mg dose of entacapone used increases the amount of ^18^F-DOPA signal in the plasma accounted for by unmetabolized ^18^F-DOPA from approximately 21 to 55%.^42^ The subjects were positioned with the orbitomeatal line parallel to the transaxial plane of the tomograph, and head position was marked and monitored via laser crosshairs and a camera. The head movement was minimized by a moulded head rest and straps. A 5-min transmission image was obtained before radiotracer injection using a 150 MBq caesium Cs 137 rotating point source to correct for attenuation and scatter.

Emission data were acquired in list mode for 95 min, rebinned into 26 time frames (comprising a 30 s background frame, four 60  s frames, three 120 s frames, three 180 s frames and finally fifteen 300 s frames), and reconstructed using a three-dimensional re-projection algorithm. Further details on the methods are reported in prior literature.^44^

### Study 2: apomorphine challenge

Twelve healthy male volunteers (mean age 26.42, s.d. 5.14; 10 right-handed) underwent ^18^F-DOPA PET scans on two separate occasions (mean days between scans=50.8 days (s.d.=109.9)). They received a 2 ml subcutaneous injection of either apomorphine or placebo (normal saline, omitted in two subjects) approximately 30 min before the start of the scan. The participants were blinded to whether they were given placebo or apomorphine. The scanning time point was selected on the basis of the pharmacokinetics to correspond to the peak period of action of apomorphine. Apomorphine has been shown to have effects on aromatic acid decarboxylase (AADC) in rat striatum within half an hour,^45^ and having biological effects for up to 120 min in people with Parkinson’s disease.^46^

#### Apomorphine dose

Five subjects received apomorphine at a dose of 0.03 mg kg^−1^ subcutaneously, based on a prior human study in Parkinson’s disease,^36^ this dose also being in line with animal work.^47^ Three subjects experienced notable autonomic side effects (nausea in one subject who had to leave the scanner and was excluded from analysis, increased blood pressure and vasodilation in two).

Consequently, the dose was decreased to improve tolerability.^22^ A dose of 0.0005 mg kg^−1^ was chosen and given to eight volunteers on the basis of behavioural and clinical studies,^39, 48^ and we hypothesized that this would not cause autonomic effects. In contrast to the higher dose, no side effects were noted with the lower apomorphine dose.

#### PET data acquisition

Approximately 150 MBq of ^18^F-DOPA was administered by bolus intravenous injection 30 s after the start of the PET imaging.

All the participants were asked not to eat or drink (except water), and refrain from alcohol for 12 h before the scan. In study 2, the imaging data were obtained on a Siemens CTI ECAT HR 962 PET scanner (Siemens, Erlanger, Germany) in three-dimensional mode. One hour before the scan, the participants received 400 mg Entacapone, a peripheral catechol-0-methyl-transferase inhibitor, and 150 mg Carbidopa, a peripheral aromatic acid decarboxylase inhibitor, to increase specific signal detection, as these compounds decrease the formation of radiolabeled metabolites that may cross the blood–brain barrier.^43^ The participants were positioned in the scanner with the orbitomeatal line parallel to the transaxial plane of the tomograph. The head position was marked and monitored and the movement was minimized using a head strap.

The PET data were acquired in 32 frames of increasing duration over the 95 min scan (frame intervals: 8 × 15 s, 3 × 60 s, 5 × 120 s frames, 16 × 300 s).

### PET analysis

For both the studies, image analysis was conducted as previously described (please see Supplementary Information).^49^ The striatal influx constant (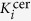, written as *K_i_* in some previous publications^41^) was calculated compared with uptake in the reference region using a graphical approach adapted for a reference tissue input function.^44^

### Statistical analysis

The percentage change in *K_i_*^cer^ was calculated as: (baseline 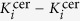 after apomorphine)/(baseline 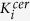) × 100. For the purpose of analysis, ‘baseline’ refers to when subjects were not given apomorphine challenge, that is, when given either placebo or no compound.

Statistical analyses were performed using SPSS (Version 21.0, IBM, Armonk, NY, USA),^50^ significance was set at *P*<0.05 (two-tailed). Normality of distribution for *K_i_*^cer^ values and percentage change was assessed using the Kolgoroff–Smirnov test. In study 1, we determined the test–retest variability of repeat ^18^F-DOPA imaging as previously reported,^44^ and determined relationships between baseline 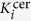 and difference between baseline and follow-up 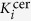 using Pearson’s correlation. In study 2, within-subject differences baseline (placebo) and stimulated (apomorphine) conditions were assessed using paired *t*-tests. A Pearson correlation coefficient was computed to assess relationships between baseline 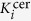 in the whole striatum and change with apomorphine.

### Testing for regression to the mean

To test whether our results may represent regression to the mean,^51^ we conducted a one-way, repeated-measures analysis of variance to assess the effect of group (test–retest or apomorphine challenge) × change (change from baseline) interaction, with the hypothesis that partial agonist effects of apomorphine (change in *K_i_*^cer^ from baseline) would distinguish the apomorphine challenge group from that of the test–retest sample. In the analysis of variance, we used Levene’s test to test the null hypothesis that the variance of the two groups was equal. We also utilized linear regression to assess whether baseline *K_i_*^cer^ and the apomorphine × baseline *K_i_*^cer^ interaction could predict second scan *K_i_*^cer^. Finally, we tested the null hypothesis that there would be no difference in means and variances of both sets of measures (test–retest data set and those given apomorphine), and the correlation between the sets of measures would be equal.^52^ For this, we converted each correlation to a *z*-score, using Fisher’s *r* to *z* transformation,^53^ then applying Steiger’s equations.^54^ A *z*-score based on the difference between the two values and variance of the difference between the two scores was obtained.^55^

## Results

### Injected activity

Mean (s.d.) injected activity for Study 1 was 147.3 (6.6) MBq (scan one) and 147.7 (2.7) MBq (scan two). There was no significant difference in activity injected between both the scans (*t*_14_=−0.16; *P*=0.88). Mean (s.d.) injected activity for Study 2 was 149.0 (9.4) MBq (scan one) and 144.9 (5.5) MBq (scan two). There was no significant difference in the activity injected between both the scans (*t*_11_=1.44; *P*=0.18).

### Study 1: reliability of ^18^F-DOPA PET imaging and the relationship between baseline dopamine synthesis capacity and change over time in untreated people

There was no significant difference between scans (mean (s.d.) *K_i_*^cer ^values: scan 1=0.014 min^−1^ (0.0015); scan 2=0.014 min^−^^1^ (0.0014)).

The interclass correlation for both the scans was 0.834, as reported previously.^44^

**Figure 1:**
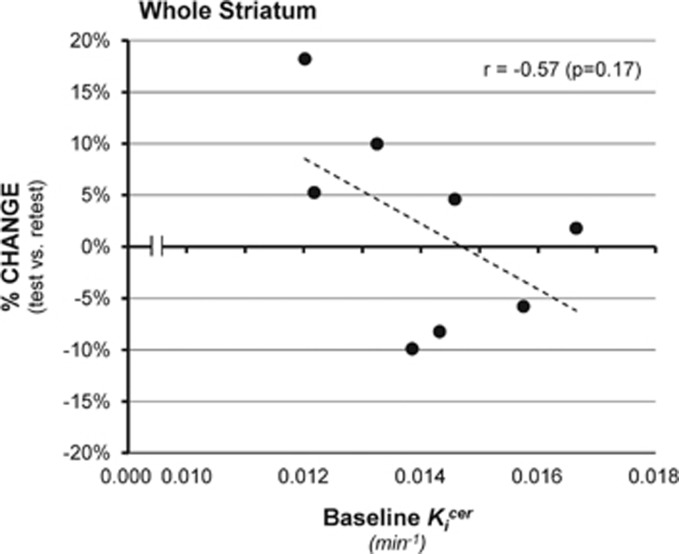
*K_i_*^cer^ at baseline and follow-up, indicating no significant difference in dopamine synthesis capacity under rest conditions.

The relationship between initial 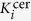 and change in 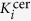 over time is shown in Figure 1. There was no significant correlation between initial 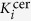 and change in *K_i_*^cer^ over time (*r*=−0.57, *n*=8, *P*=0.17; See Figure 1).

### Study 2: the effects of apomorphine on dopamine synthesis capacity

**Figure 2:**
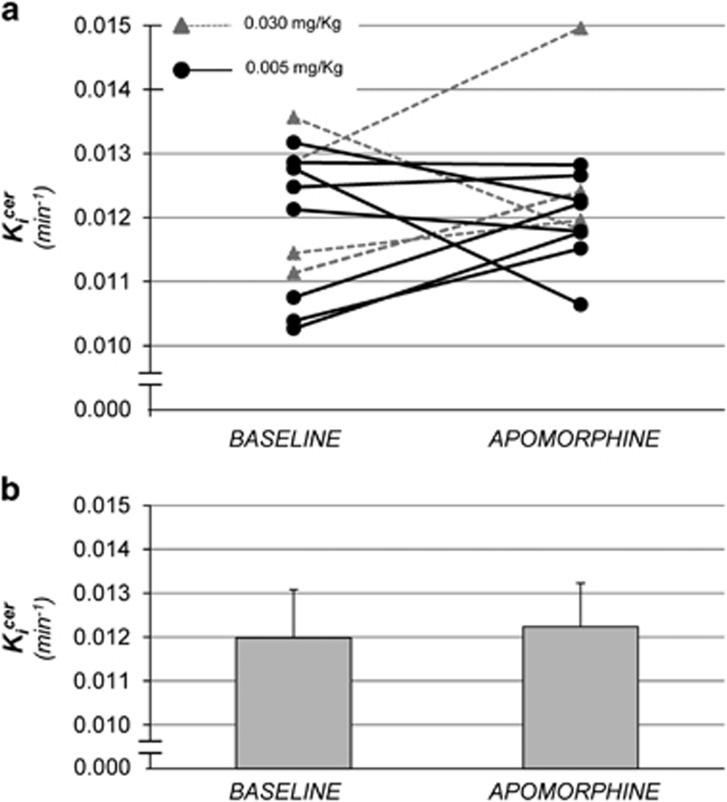
(**a**) Single-subject dopamine synthesis capacity at baseline and following apomorphine. (**b**) Mean (s.d.) dopamine synthesis capacity at baseline and following apomorphine.

The effect of apomorphine on dopamine synthesis capacity is shown in Figure 2.

There was no main effect of apomorphine on whole striatal *K_i_*^cer^ (*t*_11_=−0.71; *P*=0.49); mean (s.d.) 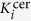 pre-apomorphine=0.0120 (0.012) min^−1^ and post-apomorphine=0.0123 (0.0010) min^−1^.

The mean (s.d.) difference (pre-apomorphine−post-apomorphine) for both doses was −0.00025 (0.0013) min^−1^, mean (s.d.) percentage change=−2.8% (1.1%).

The mean (s.d.) relative difference in dopamine synthesis capacity between baseline and post-apomorphine was 5% (11%) for 0.03 mg kg^−1^ and 2% (10%) for 0.005 mg kg^−1^.

There was no relationship between time between scans and change in *K_i_*^cer^ values (Spearman’s rho=−0.161, *P*=0.62, two-tailed).

To exclude a potential effect of apomorphine on blood flow in the reference region (cerebellum),^22^ we examined the reference region to see whether there was any change with administration of apomorphine. No difference in (as measured by the standardized uptake value at 95 min) was found (*t*_10_=0.78, *P*=0.45).

#### Relationship between baseline 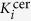 and percentage change

**Figure 3:**
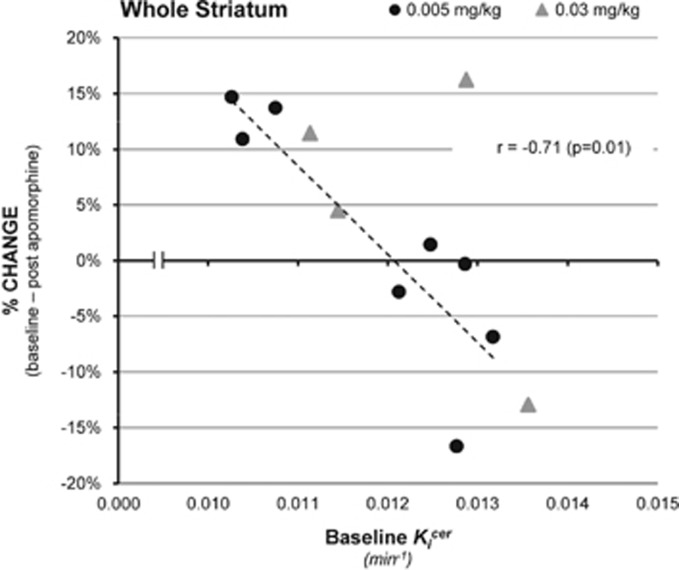
Correlation between baseline *K_i_*^cer^ and percentage change in *K_i_*^cer^ under apomorphine challenge (triangle=0.03 mg kg^−1^ dose, circle=0.005 mg kg^−1^ dose), indicating that the effect of apomorphine on dopamine synthesis capacity depends on baseline dopamine synthesis capacity.

There was a significant negative correlation between baseline value and percentage change in 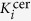 with apomorphine, *r*=−0.71, *n*=12, *P*=0.01 (two-tailed; See Figure 3). Removal of the outlier, identified in Figure 3, gave a correlation of −0.9, *n*=11, *P*<0.01.

This was larger, in absolute terms, in the subjects receiving the lower dose of apomorphine (0.005 mg kg^−1^, *r*=−0.87, *n*=8, *P*<0.01), although the difference between the two dose ranges was not statistically significant.

There was no appreciable change in effect size or *P*-value when the two subjects who did not receive placebo were excluded from the analysis (*r*=0.67, *P*=0.034, two-tailed). There was no relationship between time between scans and change in *K_i_*^cer^ values (Spearman’s rho=−0.161, *P*=0.62, two-tailed).

### Testing for regression to the mean: analysis of variance, linear regression and rate dependency bias analyses

Levene’s test indicated no statistically significant difference in variance between both the groups (*P*=0.89).

On one-way repeated-measures analysis of variance, there was a significant group (test–retest or apomorphine challenge) × change (baseline and change from baseline) interaction, F(1,18)=11.5, *P*=0.003.

We used linear regression to predict the second scan *K_i_*^cer^ based on baseline *K_i_*^cer^ and baseline–apomorphine interaction. A significant regression equation was found (F=16.6, *P*=0.001), with an adjusted *R*^2^ of 0.451, *P*=0.001. These findings were strengthened when the outlier (see Figure 3, above) was removed, leading to an adjusted *R*^2^ of 0.647, with both baseline *K_i_*^cer^ and apomorphine–baseline interaction contributing to the model (*P*=0.02 and *P*=0.01, respectively).

Using a two-tailed test of significance, a significant difference was found between the correlation in the test–retest study and apomorphine challenge study (with removal of the outlier, as above, *P*<0.01).

## Discussion

Our main finding is that the regulation of DSC by a dopamine partial agonist depends on baseline DSC. Specifically, people with relatively high baseline dopamine synthesis show a reduction, whereas those with relatively low baseline values show an increase in DSC. This finding is consistent with apomorphine stabilizing DSC as a partial agonist. Partial dopamine receptor agonists increase dopamine receptor signalling if dopamine levels are low and decrease dopamine receptor signalling when dopamine levels are high as they compete with dopamine and given their lower intrinsic activity the net output is lower than dopamine *per se*. Moreover, we did not see a relationship between baseline dopamine synthesis capacity and change over time in our test–retest study, where there was no apomorphine administration, indicating that the effect seen with apomorphine is unlikely to be explained by regression to the mean.

### Comparison with other imaging studies

Our findings extend those of an L-11C-DOPA study of rhesus monkeys, which also found a strong negative relationship between baseline *K_i_* value and apomorphine induced change in *K_i_* value (*r*=−0.93), to show this in humans.^35^ Our results are also similar to findings from an L-11C-DOPA study carried out in people with early- and late-stage Parkinson’s disease.^37^ Ekesbo *et al.*^37^ found a significant effect of stage of Parkinson’s disease on the effect of apomorphine such that patients with early-stage disease showed a reduction, whereas patients with late stage showed an increase in DSC in absolute terms, though this was not statistically significant. The patients with early-stage disease who showed a reduction with apomorphine had *K_i_* values similar to those in our subjects who showed a reduction with apomorphine, whereas those with late-stage disease had low *K_i_* values, lower in absolute terms than in our subjects, who showed an increase. Our findings thus extend these findings in non-human primates and Parkinson’s disease to indicate that the normal regulation of DSC also depends on baseline dopaminergic function.

### Dopaminergic responses dependent on baseline dopamine

In addition to the studies discussed above, several other lines of evidence show this dependency on baseline dopamine function. In the Parkinson’s disease literature, animal and human studies have shown using functional magnetic resonance imaging increased the blood oxygen level-dependent activation in dopamine-deficient states, following apomorphine infusion.^56, 57, 58, 59, 60^ A human PET study, in early and advanced Parkinson’s disease, showed that response to L-DOPA was dependent on baseline status, with those with mild PD showing decreased striatal influx of L-DOPA, and those with advanced PD showing an increase in striatal L-DOPA uptake.^61^ A recent rodent study, examined the effects of aripiprazole (a partial dopamine agonist) on dopamine synthesis in rodents, finding that its effects on presynaptic D_2_ autoreceptors was either as an agonist or antagonist, depending on whether the experimental condition was of low or high dopaminergic tone.^62^ This finding has also been seen in healthy volunteers given the partial dopamine agonist aripiprazole or the dopamine antagonists haloperidol or risperidone.^63, 64, 65^

### Mechanism of action

Our findings are consistent with evidence that apomorphine acts as a partial agonist at dopamine D_2_ receptors.^29^ This indicates that in healthy volunteers, with relatively high DSC and presumed relatively low-tonic auto-inhibition by dopamine, apomorphine will act as an agonist at autoreceptors, to decrease DSC.

Conversely, in healthy volunteers with relatively low DSC and presumed relatively high tonic auto-inhibition by dopamine, apomorphine will compete with dopamine to occupy the autoreceptor. As apomorphine lacks the full agonist action, it will decrease the net functional agonist effect when it displaces dopamine, thereby causing increased DSC. This is in line with competitive binding studies.^29^ The data from CHO-expressed recombinant human D_2S_, D_2L_, D_3_ and D_4_ receptors measuring the influence of apomorphine on [^35^S]GTPγS binding both alone and in combination with dopamine points to it having partial agonist effects, as noted above.^29^ Nevertheless, it is important to note that partial agonism has not been shown in intact preparations, where apomorphine is generally found to behave as a full agonist, so this interpretation of our findings should be considered tentative at this stage.

### Limitations

Our study was not intended to examine dose effects. Consequently, the sample size for the higher dose of apomorphine was small, which means that our analysis of dose effects lacks power and should be considered preliminary. It would be useful to use a wider dose range in future work to definitively test the dose effects. Our study design does not prove a causal relationship between baseline DSC and response to dopamine agonism. This could be done in preclinical experiments using optogenetic or other techniques to alter baseline DSC.

The test–retest comparison group had a longer duration of time between scans than the apomorphine challenge group. Despite this length of time, however, there was good test–retest reliability between scans in this group, and no evidence of regression to the mean. Nevertheless, it would be useful for future studies to test this over the same duration as used in the challenge group. It should also be noted that a few of the participants in the test–retest study showed large absolute differences, of −10% to 20% although at the group level, the variability was much less. This likely reflects subject and scan acquisition-related variability, such as small movements of the subject during one scan session. However, this individual level variability would reduce our power to detect the relationship between baseline dopamine synthesis capacity and change under apomorphine. As such, it does not account for our findings, and could reduce the strength of the relationship.

### Pharmacological considerations

Other considerations include possible effects of apomorphine on blood flow in the reference region (cerebellum), and the possibility that apomorphine could have effects on the peripheral elimination or metabolism of DOPA. However, these are unlikely to explain our results for the following reasons. First, we did not find differences in DOPA uptake in the reference region pre- or post-apomorphine. The consistency of ^18^F-DOPA activity in cerebellum at baseline and after apomorphine that we found would suggest that apomorphine is not having an effect on blood flow (please see Figure 1 and Supplementary Material). Nevertheless, further investigation using arterial blood sampling is needed to definitively exclude any significant alteration on blood flow by apomorphine at the dosage used in this study.

Although we were unable to test whether apomorphine would affect metabolism of DOPA in our sample, previous animal literature has failed to find any effect.^35^ Assuming an effect of apomorphine on blood flow, as shown in preclinical studies,^66^ this would have been uni-directional (either an increase or decrease compared with baseline conditions) and in contrast to our findings, which suggest bidirectional modulation of dopamine synthesis. Two subjects did not receive placebo during baseline scan, which may introduce an expectancy effect. However, taking these two subjects out of the analysis did not significantly change magnitude of effect size or *P*-value, as shown above. We accept that, in the subjects who reported side effects with apomorphine, breaking of the blind could have occurred. This could have potentially affected dopamine release in these individuals.^67^ It should be noted, however, that in those people receiving apomorphine at the lower dose (with no side effects) the results were unchanged, and magnitude of correlation was larger.

It is conceivable that, as *K_i_* is calculated from *K_i_*=K1 × k3(k2+k3), which is a rectangular hyperbola, if *K_i_* is higher, a change in k3 will have different effects on *K_i_* than when Ki is lower. We therefore ran a simulation using standard values for K1, k2 and k3.^68^ We do not know the real change in k3 but around control values the relationship between *K_i_* and k3 is not exactly linear. Assuming a +30% change in k3, this would translate into a +33% change for subjects with the largest *K_i_* and in a +40% change for those with the lowest *K_i_*. Therefore, it is unlikely that our results reflect a statistical artefact. It could also be hypothesized that the conversion rate, k3, may be saturable, and therefore one would not see a change in those with higher baseline *K_i_*^cer^. However our results argue against this. In our study, similar changes were seen in those with both high and low baseline *K_i_*^cer^, in keeping with other pharmacological studies using the F-DOPA ligand, which have shown a decrease in *K_i_*^cer^ in those with relatively higher baseline *K_i_*^cer^.^63, 69^ Furthermore, in normal conditions, AADC is not the rate-limiting step in dopamine synthesis and apomorphine at the doses used in the study is not known to directly influence AADC levels, but modifies it functionally via dopamine receptors.^70^ In these conditions, tracer quantities of ^18^F-DOPA will not saturate AADC. However, it should be noted that AADC may be saturable when high doses of L-DOPA are given, for example, as a treatment for Parkinson’s disease. Finally, it is worth examining the possible acute and chronic effects of apomorphine. Animal work suggests differential effects of apomorphine acutely (with a decrease in striatal dopamine turnover with acute treatment, and an increase with chronic treatment).^71^ In humans, chronic treatment with D_2_ partial agonists with high intrinsic activity, aimed at selectively engaging the autoreceptors, has not been effective in treating schizophrenia.^72^ The main reason attributed has been desensitization of D_2_ autoreceptors. However, animal literature is mixed in this regard.^73, 74^

### Therapeutic implications

Our finding that low-dose apomorphine has a stabilizing effect on dopaminergic function has implications for the treatment of a number of disorders in addition to Parkinson’s disease. In schizophrenia, where DSC is predominantly elevated,^13, 14^ our findings indicate that a low level of dopamine agonism may act to reduce elevated DSC. The low dose used suggests there will not be significant postsynaptic effects, reducing the risk of exacerbating psychosis or other side effects. Indeed, there is evidence of efficacy of apomorphine in schizophrenia, although initial positive results^75^ were not replicated,^72^ possibly because doses used in some studies were higher (up to 6 mg) than those we used, as well as desensitization, as noted above. Dopamine agonism could also have a role in substance dependence (particularly stimulant dependence) to stabilize dopaminergic neurotransmission by potentially providing tonic dopaminergic stimulation and reducing surges in dopamine associated with drug-related cues. Our findings support studies in these conditions to determine the effect of dopaminergic stimulation on dopamine function.

## References

[bib1] Carlsson A, Lindqvist M, Magnusson T. 3,4-Dihydroxyphenylalanine and 5-hydroxytryptophan as reserpine antagonists. Nature 1957; 180: 1200–1200. 10.1038/1801200a0 13483658

[bib2] Yokel RA, Wise RA. Increased lever pressing for amphetamine after pimozide in rats: implications for a dopamine theory of reward. Science 1975; 187: 547–549. 1114313 10.1126/science.1114313

[bib3] Berridge KC, Robinson TE. What is the role of dopamine in reward: hedonic impact, reward learning, or incentive salience? Brain Res Rev 1998; 28: 309–369. 9858756 10.1016/s0165-0173(98)00019-8

[bib4] Mehta SH, Morgan JC, Sethi KD. Sleep disorders associated with Parkinson’s disease: role of dopamine, epidemiology, and clinical scales of assessment. CNS Spectr 2008; 13: 6–11. 10.1017/s1092852900017260 18323761

[bib5] Barbeau A. The pathogenesis of Parkinson’s disease: a new hypothesis. Can Med Assoc J 1962; 87: 802. 13966498 PMC1849683

[bib6] Siessmeier T, Kienast T, Wrase J, Larsen JL, Braus DF, Smolka MN et al. Net influx of plasma 6-[18 F]fluoro-l-DOPA (FDOPA) to the ventral striatum correlates with prefrontal processing of affective stimuli. Eur J Neurosci 2006; 24: 305–313. 16882026 10.1111/j.1460-9568.2006.04903.x

[bib7] Schlagenhauf F, Rapp MA, Huys QJM, Beck A, Wüstenberg T, Deserno L et al. Ventral striatal prediction error signaling is associated with dopamine synthesis capacity and fluid intelligence. Hum Brain Mapp 2013; 34: 1490–1499. 22344813 10.1002/hbm.22000 PMC3731774

[bib8] Ernst M, Zametkin AJ, Matochik JA, Jons PH, Cohen RM. DOPA decarboxylase activity in attention deficit hyperactivity disorder adults. A [fluorine-18]fluorodopa positron emission tomographic study. J Neurosci 1998; 18: 5901–5907. 9671677 10.1523/JNEUROSCI.18-15-05901.1998 PMC6793062

[bib9] Lotharius J, Brundin P. Pathogenesis of parkinson’s disease: dopamine, vesicles and α-synuclein. Nat Rev Neurosci 2002; 3: 932–942. 12461550 10.1038/nrn983

[bib10] van Rossum JM. The significance of dopamine-receptor blockade for the mechanism of action of neuroleptic drugs. Arch Int Pharmacodyn Thérapie 1966; 160: 492–494. 5954044

[bib11] Howes OD, Kapur S. The dopamine hypothesis of schizophrenia: version III—the final common pathway. Schizophr Bull 2009; 35: 549–562. 19325164 10.1093/schbul/sbp006 PMC2669582

[bib12] Koob GF, Bloom FE. Cellular and molecular mechanisms of drug dependence. Science 1988; 242: 715–723. 2903550 10.1126/science.2903550

[bib13] Hietala J, Syvälahti E, Vuorio K, Räkköläinen V, Bergman J, Haaparanta M et al. Presynaptic dopamine function in striatum of neuroleptic-naive schizophrenic patients. Lancet 1995; 346: 1130. 7475604 10.1016/s0140-6736(95)91801-9

[bib14] Howes OD, Kambeitz J, Kim E, Stahl D, Slifstein M, Abi-Dargham A et al. The nature of dopamine dysfunction in schizophrenia and what this means for treatment: meta-analysis of imaging studies. Arch Gen Psychiatry 2012; 69: 776–786. 22474070 10.1001/archgenpsychiatry.2012.169 PMC3730746

[bib15] Egerton A, Chaddock CA, Winton-Brown TT, Bloomfield MAP, Bhattacharyya S, Allen P et al. Presynaptic striatal dopamine dysfunction in people at ultra-high risk for psychosis: findings in a second cohort. Biol Psychiatry 2013; 74: 106–112. 23312565 10.1016/j.biopsych.2012.11.017

[bib16] Howes OD, Bose SK, Turkheimer F, Valli I, Egerton A, Valmaggia LR et al. Dopamine synthesis capacity before onset of psychosis: a prospective [18 F]-DOPA PET imaging study. Am J Psychiatry 2011; 168: 1311–1317. 21768612 10.1176/appi.ajp.2011.11010160 PMC3682447

[bib17] Ludolph AG, Kassubek J, Schmeck K, Glaser C, Wunderlich A, Buck AK et al. Dopaminergic dysfunction in attention deficit hyperactivity disorder (ADHD), differences between pharmacologically treated and never treated young adults: a 3,4-dihdroxy-6-[18 F]fluorophenyl-l-alanine PET study. Neuroimage 2008; 41: 718–727. 18424180 10.1016/j.neuroimage.2008.02.025

[bib18] Dhawan V, Ma Y, Pillai V, Spetsieris P, Chaly T, Belakhlef A et al. Comparative analysis of striatal FDOPA uptake in Parkinson’s disease: ratio method versus graphical approach. J Nucl Med 2002; 43: 1324–1330. 12368370

[bib19] Bloomfield MAP, Morgan CJA, Egerton A, Kapur S, Curran HV, Howes OD. Dopaminergic function in cannabis users and its relationship to cannabis-induced psychotic symptoms. Biol Psychiatry 2014; 75: 470–478. 23820822 10.1016/j.biopsych.2013.05.027

[bib20] Kumakura Y, Gjedde A, Caprioli D, Kienast T, Beck A, Plotkin M et al. Increased turnover of dopamine in caudate nucleus of detoxified alcoholic patients. PLoS One 2013; 8: e73903. 24040111 10.1371/journal.pone.0073903 PMC3770672

[bib21] Salokangas RK, Vilkman H, Ilonen T, Taiminen T, Bergman J, Haaparanta M et al. High levels of dopamine activity in the basal ganglia of cigarette smokers. Am J Psychiatry 2000; 157: 632–634. 10739427 10.1176/appi.ajp.157.4.632

[bib22] Langer SZ. Presynaptic regulation of the release of catecholamines. Pharmacol Rev 1980; 32: 337–362. 6267618

[bib23] Westerink BHC, de Vries JB. On the mechanism of neuroleptic induced increase in striatal dopamine release: brain dialysis provides direct evidence for mediation by autoreceptors localized on nerve terminals. Neurosci Lett 1989; 99: 197–202. 2526308 10.1016/0304-3940(89)90289-9

[bib24] Freeze BS, Kravitz AV, Hammack N, Berke JD, Kreitzer AC. Control of basal ganglia output by direct and indirect pathway projection neurons. J Neurosci 2013; 33: 18531–18539. 24259575 10.1523/JNEUROSCI.1278-13.2013 PMC3834057

[bib25] Shi W-X, Pun C-L, Smith PL, Bunney BS. Endogenous DA-mediated feedback inhibition of DA neurons: involvement of both D1- and D2-like receptors. Synapse 2000; 35: 111–119. 10611636 10.1002/(SICI)1098-2396(200002)35:2<111::AID-SYN3>3.0.CO;2-7

[bib26] Saklayen SS, Mabrouk OS, Pehek EA. Negative feedback regulation of nigrostriatal dopamine release: mediation by striatal D1 receptors. J Pharmacol Exp Ther 2004; 311: 342–348. 15175419 10.1124/jpet.104.067991

[bib27] Santiago M, Westerink BHC. The regulation of dopamine release from nigrostriatal neurons in conscious rats: the role of somatodendritic autoreceptors. Eur J Pharmacol 1991; 204: 79–85. 1687125 10.1016/0014-2999(91)90838-h

[bib28] Stibe CMH, Kempster PA, Lees AJ, Stern GM. Subcutaneous apomorphine in parkinsonian on-off oscillations. Lancet 1988; 331: 403–406. 10.1016/s0140-6736(88)91193-2 2893200

[bib29] Newman-Tancredi A, Cussac D, Audinot V, Nicolas J-P, Ceuninck FD, Boutin J-A et al. Differential actions of antiparkinson agents at multiple classes of monoaminergic receptor. II. Agonist and antagonist properties at subtypes of dopamine D2-like receptor and α1/α2-adrenoceptor. J Pharmacol Exp Ther 2002; 303: 805–814. 12388667 10.1124/jpet.102.039875

[bib30] Gainetdinov RR, Sotnikova TD, Grekhova TV, Rayevsky KS. *In vivo* evidence for preferential role of dopamine D3 receptor in the presynaptic regulation of dopamine release but not synthesis. Eur J Pharmacol 1996; 308: 261–269. 8858296 10.1016/0014-2999(96)00300-7

[bib31] Kehr W, Carlsson A, Lindqvist M. Catecholamine synthesis in rat brain after axotomy: interaction between apomorphine and haloperidol. Naunyn Schmiedebergs Arch Pharmacol 1977; 297: 111–117. 193050 10.1007/BF00499920

[bib32] Walthers JR, Roth RH. Dopaminergic neurons: an *in vivo* system for measuring drug interactions with presynaptic receptors. Naunyn Schmiedebergs Arch Pharmacol 1976; 296: 5–14. 13315 10.1007/BF00498834

[bib33] White FJ, Wang RY. A10 dopamine neurons: role of autoreceptors in determining firing rate and sensitivity to dopamine agonists. Life Sci 1984; 34: 1161–1170. 6708722 10.1016/0024-3205(84)90088-2

[bib34] Di Chiara G, Porceddu ML, Vargiu L, Argiolas A, Gessa GL. Evidence for dopamine receptors mediating sedation in the mouse brain. Nature 1976; 264: 564–567. 1004597 10.1038/264564a0

[bib35] Torstenson R, Hartvig P, Långström B, Bastami S, Antoni G, Tedroff J. Effect of apomorphine infusion on dopamine synthesis rate relates to dopaminergic tone. Neuropharmacology 1998; 37: 989–995. 9833628 10.1016/s0028-3908(98)00085-9

[bib36] de La Fuente-Fernández R, Lim AS, Sossi V, Holden JE, Calne DB, Ruth TJ et al. Apomorphine-induced changes in synaptic dopamine levels: positron emission tomography evidence for presynaptic inhibition. J Cereb Blood Flow Metab 2001; 21: 1151–1159. 11598492 10.1097/00004647-200110000-00003

[bib37] Ekesbo A, Rydin E, Torstenson R, Sydow O, Låengström B, Tedroff J. Dopamine autoreceptor function is lost in advanced Parkinson’s disease. Neurology 1999; 52: 120–125. 9921858 10.1212/wnl.52.1.120

[bib38] Pucak ML, Grace AA. Partial dopamine depletions result in an enhanced sensitivity of residual dopamine neurons to apomorphine. Synapse 1991; 9: 144–155. 1821486 10.1002/syn.890090209

[bib39] Lal S, Tesfaye Y, Thavundayil JX, Thompson TR, Kiely ME, Nair NPV et al. Apomorphine: clinical studies on erectile impotence and yawning. Prog Neuropsychopharmacol Biol Psychiatry 1989; 13: 329–339. 2748870 10.1016/0278-5846(89)90122-x

[bib40] First MB. Structured Clinical Interview for the DSM (SCID). In:*The Encyclopedia of Clinical Psychology*. John Wiley & Sons, 2014 Available athttp://onlinelibrary.wiley.com/doi/10.1002/9781118625392.wbecp351/abstract (accessed 19 March 2015).

[bib41] Howes OD, Montgomery AJ, Asselin MC, Murray RM, Valli I, Tabraham P et al. Elevated striatal dopamine function linked to prodromal signs of schizophrenia. Arch Gen Psychiatry 2009; 66: 13. 19124684 10.1001/archgenpsychiatry.2008.514

[bib42] Sawle GV, Burn DJ, Morrish PK, Lammertsma AA, Snow BJ, Luthra S et al. The effect of entacapone (OR-611) on brain [18 F]-6- L-fluorodopa metabolism: implications for levodopa therapy of Parkinson’s disease. Neurology 1994; 44: 1292–1297. 8035933 10.1212/wnl.44.7.1292

[bib43] Cumming P, Léger GC, Kuwabara H, Gjedde A. Pharmacokinetics of plasma 6-[18 F]fluoro-L-3,4-dihydroxyphenylalanine ([18 F]Fdopa) in humans. J Cereb Blood Flow Metab 1993; 13: 668–675. 8314919 10.1038/jcbfm.1993.85

[bib44] Egerton A, Demjaha A, McGuire P, Mehta MA, Howes OD. The test-retest reliability of 18 F-DOPA PET in assessing striatal and extrastriatal presynaptic dopaminergic function. Neuroimage 2010; 50: 524–531. 20034580 10.1016/j.neuroimage.2009.12.058 PMC4096947

[bib45] Zhu M-Y, Juorio AV, Paterson IA, Boulton AA. Regulation of aromatic l-amino acid decarboxylase in rat striatal synaptosomes: effects of dopamine receptor agonists and antagonists. Br J Pharmacol 1994; 112: 23–30. 7913379 10.1111/j.1476-5381.1994.tb13023.x PMC1910301

[bib46] Neef C, van Laar T. Pharmacokinetic-pharmacodynamic relationships of apomorphine in patients with Parkinson’s disease. Clin Pharmacokinet 1999; 37: 257–271. 10511920 10.2165/00003088-199937030-00004

[bib47] Grace AA, Bunney BS. Low doses of apomorphine elicit two opposing influences on dopamine cell electrophysiology. Brain Res 1985; 333: 285–298. 3995296 10.1016/0006-8993(85)91582-3

[bib48] Levy MI, Davis BM, Mohs RC, Kendler KS, Mathé AA, Trigos G et al. Apomorphine and schizophrenia. Treatment, CSF, and neuroendocrine responses. Arch Gen Psychiatry 1984; 41: 520–524. 6372737 10.1001/archpsyc.1984.01790160106014

[bib49] Bloomfield MAP, Pepper F, Egerton A, Demjaha A, Tomasi G, Mouchlianitis E et al. Dopamine function in cigarette smokers: an [^18^F]-DOPA PET study. Neuropsychopharmacology 2014; 39: 2397–2404. 24718373 10.1038/npp.2014.87 PMC4138749

[bib50] IBM. Downloading IBM SPSS Statistics 21 - United States. 2012. Available at http://www-01.ibm.com/support/docview.wss?uid=swg24032236 (accessed 3 June 2015).

[bib51] Barnett AG, van der Pols JC, Dobson AJ. Regression to the mean: what it is and how to deal with it. Int J Epidemiol 2005; 34: 215–220. 15333621 10.1093/ije/dyh299

[bib52] Lee IA, Preacher K. Calculation for the test of the difference between two dependent correlations with one variable in common, 2013. Available athttp://quantpsy.org.

[bib53] SAMPLE CDFAS. Correlation coefficients covering the cases (i)‘The frequency distribution of the values of the correlation coefficient in samples from an indefinitely large population,’ Biometrika, Vol. 10, pp. 507v521, 1915. Here the method of defining the sample by the coordinates of. 1921. Available at http://www.ssnpstudents.com/wp/wp-content/uploads/2015/02/fisher.pdf4_.pdf (accessed 14 August 2015).

[bib54] Steiger JH. Tests for comparing elements of a correlation matrix. Psychol Bull 1980; 87: 245–251.

[bib55] Teicher MH, Polcari A, Anderson CM, Andersen SL, Lowen SB, Navalta CP. Rate dependency revisited: understanding the effects of methylphenidate in children with attention deficit hyperactivity disorder. J Child Adolesc Psychopharmacol 2003; 13: 41–51. 12804125 10.1089/104454603321666180

[bib56] Zhang Z, Andersen AH, Avison MJ, Gerhardt GA, Gash DM. Functional MRI of apomorphine activation of the basal ganglia in awake rhesus monkeys. Brain Res 2000; 852: 290–296. 10678755 10.1016/s0006-8993(99)02243-x

[bib57] Zhang Z, Andersen A, Grondin R, Barber T, Avison R, Gerhardt G et al. Pharmacological MRI mapping of age-associated changes in basal ganglia circuitry of awake rhesus monkeys. Neuroimage 2001; 14: 1159–1167. 11697947 10.1006/nimg.2001.0902

[bib58] Zhang Z, Andersen AH, Ai Y, Loveland A, Hardy PA, Gerhardt GA et al. Assessing nigrostriatal dysfunctions by pharmacological MRI in parkinsonian rhesus macaques. Neuroimage 2006; 33: 636–643. 16949305 10.1016/j.neuroimage.2006.07.004

[bib59] Nguyen TV, Brownell AL, Iris Chen YC, Livni E, Coyle JT, Rosen BR et al. Detection of the effects of dopamine receptor supersensitivity using pharmacological MRI and correlations with PET. Synapse 2000; 36: 57–65. 10700026 10.1002/(SICI)1098-2396(200004)36:1<57::AID-SYN6>3.0.CO;2-K

[bib60] Passamonti L, Salsone M, Toschi N, Cerasa A, Giannelli M, Chiriaco C et al. Dopamine-transporter levels drive striatal responses to apomorphine in Parkinson’s disease. Brain Behav 2013; 3: 249–262. 23785657 10.1002/brb3.115 PMC3683285

[bib61] Torstenson R, Hartvig P, Långström B, Westerberg G, Tedroff J. Differential effects of levodopa on dopaminergic function in early and advanced Parkinson’s disease. Ann Neurol 1997; 41: 334–340. 9066354 10.1002/ana.410410308

[bib62] Ma GF, Raivio N, Sabrià J, Ortiz J. Agonist and antagonist effects of aripiprazole on D_2_-like receptors controlling rat brain dopamine synthesis depend on the dopaminergic tone. Int J Neuropsychopharmacol 2014; 18: pyu046. 25522390 10.1093/ijnp/pyu046 PMC4360222

[bib63] Ito H, Takano H, Arakawa R, Takahashi H, Kodaka F, Takahata K et al. Effects of dopamine D2 receptor partial agonist antipsychotic aripiprazole on dopamine synthesis in human brain measured by PET with L-[β-11C]DOPA. PLoS One 2012; 7: e46488. 23029533 10.1371/journal.pone.0046488 PMC3460902

[bib64] Ito H, Takano H, Takahashi H, Arakawa R, Miyoshi M, Kodaka F et al. Effects of the antipsychotic risperidone on dopamine synthesis in human brain measured by positron emission tomography with l-[β-11C]DOPA: a stabilizing effect for dopaminergic neurotransmission? J Neurosci 2009; 29: 13730–13734. 19864585 10.1523/JNEUROSCI.4172-09.2009 PMC6665007

[bib65] Vernaleken I, Kumakura Y, Buchholz H-G, Siessmeier T, Hilgers R-D, Bartenstein P et al. Baseline [18 F]-FDOPA kinetics are predictive of haloperidol-induced changes in dopamine turnover and cognitive performance: a positron emission tomography study in healthy subjects. Neuroimage 2008; 40: 1222–1231. 18262797 10.1016/j.neuroimage.2007.12.045

[bib66] McCulloch J, Kelly PA, Ford I. Effect of apomorphine on the relationship between local cerebral glucose utilization and local cerebral blood flow (with an appendix on its statistical analysis). J Cereb Blood Flow Metab 1982; 2: 487–499. 7142312 10.1038/jcbfm.1982.56

[bib67] Lidstone SC, Schulzer M, Dinelle K, Mak E, Sossi V, Ruth TJ et al. Effects of expectation on placebo-induced dopamine release in Parkinson disease. Arch Gen Psychiatry 2010; 67: 857–865. 20679593 10.1001/archgenpsychiatry.2010.88

[bib68] Schiepers C, Chen W, Cloughesy T, Dahlbom M, Huang S-C. 18 F-FDOPA kinetics in brain tumors. J Nucl Med 2007; 48: 1651–1661. 17873130 10.2967/jnumed.106.039321

[bib69] Gründer G, Vernaleken I, Müller MJ, Davids E, Heydari N, Buchholz HG et al. Subchronic haloperidol downregulates dopamine synthesis capacity in the brain of schizophrenic patients *in vivo*. Neuropsychopharmacology 2003; 28: 787–794. 12655326 10.1038/sj.npp.1300103

[bib70] Zhu MY, Juorio AV, Paterson IA, Boulton AA. Regulation of aromatic L-amino acid decarboxylase in rat striatal synaptosomes: effects of dopamine receptor agonists and antagonists. Br J Pharmacol 1994; 112: 23–30. 7913379 10.1111/j.1476-5381.1994.tb13023.x PMC1910301

[bib71] Rowlett J. Neurochemical and behavioral effects of acute and chronic treatment with apomorphine in rats. Neuropharmacology 1991; 30: 191–197. 1903190 10.1016/0028-3908(91)90203-n

[bib72] Tamminga CA. Partial dopamine agonists in the treatment of psychosis. J Neural Transm (Vienna) 2002; 109: 411–420. 11956961 10.1007/s007020200033

[bib73] Nickolson VJ, van Riezen H, van Delft AM. Response changes after repeated low apomorphine: dopamine autoreceptor desensitization or learning? Psychopharmacology (Berl) 1984; 83: 188–193. 6431472 10.1007/BF00429733

[bib74] Jeziorski M, White FJ. Dopamine agonists at repeated ‘autoreceptor-selective’ doses: effects upon the sensitivity of A10 dopamine autoreceptors. Synapse 1989; 4: 267–280. 2603146 10.1002/syn.890040403

[bib75] Tamminga CA, Schaffer MH, Smith RC, Davis JM. Schizophrenic symptoms improve with apomorphine. Science 1978; 200: 567–568. 347574 10.1126/science.347574

