# Correction: Regulation of dopaminergic function: an [^18^F]-DOPA PET apomorphine challenge study in humans

**DOI:** 10.1038/s41398-018-0187-6

**Published:** 2018-08-31

**Authors:** S. Jauhar, M. Veronese, M. Rogdaki, M. Bloomfield, S. Natesan, F. Turkheimer, S. Kapur, O. D. Howes

**Affiliations:** 1 Department of Psychosis Studies, Institute of Psychiatry, Psychology and Neuroscience, King’s College, London, UK; 2 Centre for Neuroimaging Sciences, Institute of Psychiatry, Psychology and Neuroscience, King’s College, London, UK; 3 MRC London Institute of Medical Sciences, London, UK; 4 Institute of Clinical Sciences, Department of Medicine, Imperial College London, London, UK

Correction to: *Translational Psychiatry* ; 10.1038/tp.2016.270; published online 07 February 2017

This article was originally published under a CC BY-NC-ND 4.0 license, but has now been made available under a CC BY 4.0 license. The PDF and HTML versions of the article have been modified accordingly.

