# Correction: Regulation of dopaminergic function: An [^18^F]-DOPA PET apomorphine challenge study in humans

**DOI:** 10.1038/s41398-019-0501-y

**Published:** 2019-06-11

**Authors:** S Jauhar, M Veronese, M Rogdaki, M Bloomfield, S Natesan, F Turkheimer, S Kapur, O D Howes

**Affiliations:** 1 Department of Psychosis Studies, Institute of Psychiatry, Psychology and Neuroscience, King’s College, London, UK; 2 Centre for Neuroimaging Sciences, Institute of Psychiatry, Psychology and Neuroscience, King’s College, London, UK; 3 MRC London Institute of Medical Sciences, London, UK; 4 Institute of Clinical Sciences, Department of Medicine, Imperial College London, London, UK

**Correction to: Translational Psychiatry**

10.1038/tp.2016.270 Published online 07 February 2017

The author noticed and would like to amend a few errors in the Original Article:

1. **Statistical analysis, p2** “The percentage change in *Ki*cer was calculated as: (baseline Ki cer-Ki cer after apomorphine)/(baseline Ki cer) × 100. For the purpose of analysis, ‘baseline’ refers to when subjects were not given apomorphine challenge, that is, when given either placebo or no compound.” **This should have read as** “The percentage change in *Ki*cer was calculated as: (**Ki cer after apomorphine-baseline Ki cer)/(baseline Ki cer)** **×** **100**. For the purpose of analysis, ‘baseline’ refers to when subjects were not given apomorphine challenge, that is, when given either placebo or no compound.”
2. **Figure 3:** The legend on the y axis states % change (Baseline-post-apomorphine), **which should read as baseline vs post-apomorphine**. This was in the original submission, though we fully accept the error when this went through copy editing.
3. **P3** states, e interclass correlation for both the scans was 0.834, as reported previously. **This should read as 0.843**.
4. **P3**, it reads “There was no appreciable change in effect size or *P*-value when the two subjects who did not receive placebo were excluded from the analysis (*r* = 0.67, *P* = 0.034, two-tailed).” **It should read as −0.67, ie., a negative correlation**.

