# Supplementary material for: Regulation of dopaminergic function: an [18F]-DOPA PET apomorphine challenge study in humans
Source: Transl Psychiatry. 2017 Feb 7;7(2):e1027–. doi: 10.1038/tp.2016.270 (PMC5438020; doi:10.1038/tp.2016.270)
Supplement: Supplementary Information [file tp2016270x1.pdf]

Supplemetary information

Correction for head movement during the scan was performed by denoising the non-attenuation-corrected dynamic images using a level 2, order 64 Battle- Lemarie wavelet filter. Frames were realigned to a single 'reference' frame, acquired 20 min post-injection, employing a mutual information algorithm [1,2]. The transformation parameters were then applied to the corresponding attenuated-corrected dynamic images, creating a movement-corrected dynamic image, which was used in the analysis. Realigned frames were then summated to create an individual motion-corrected reference map for the brain tissue segmentation. SPM5 (http://www.fil.ion.ucl.ac.uk/spm) was used to normalize a tracer-specific ([18F]-DOPA) template [3,4] together with the HamNet probabilistic brain atlas [5] both in the same space to each individual PET summation image. The HamNet brain atlas was used to identify the whole striatum and the cerebellar (reference) region[6]. The striatal influx constant (K_i^{cer}, K_i in some previous publications) [4] was calculated compared with uptake in the reference region using a graphical approach adapted for reference tissue input function [3].

References

. 1 StudholmeC,HillDL,HawkesDJ.Automated3-DregistrationofMRandCT images of the head. Med Image Anal 1996; 1: 163–175.

. 2 TurkheimerFE,BrettM,VisvikisD,CunninghamVJ.Multiresolutionanaly sis of emission tomography images in the wavelet domain. J Cereb Blood Flow Metab Off J Int Soc Cereb Blood Flow Metab 1999; 19: 1189–1208.

. 3 EgertonA,DemjahaA,McGuireP,MehtaMA,HowesOD.Thetest-retest reliability of 18F-DOPA PET in assessing striatal and extrastriatal presynaptic dopaminergic function. NeuroImage 2010; 50: 524–531.

. 4 HowesOD,MontgomeryAJ,AsselinMC,MurrayRM,ValliI,TabrahamPetal . Elevated striatal dopamine function linked to prodromal signs of schizophrenia. Arch Gen Psychiatry 2009; 66: 13.
