# Supplementary material for: Regulation of dopaminergic function: an [18F]-DOPA PET apomorphine challenge study in humans
Source: Transl Psychiatry. 2017 Feb 7;7(2):e1027–. doi: 10.1038/tp.2016.270 (PMC5438020; doi:10.1038/tp.2016.270)

Comparison of ^{18}F-DOPA kinetic activity in cerebellum at baseline and following apomorphine challenge

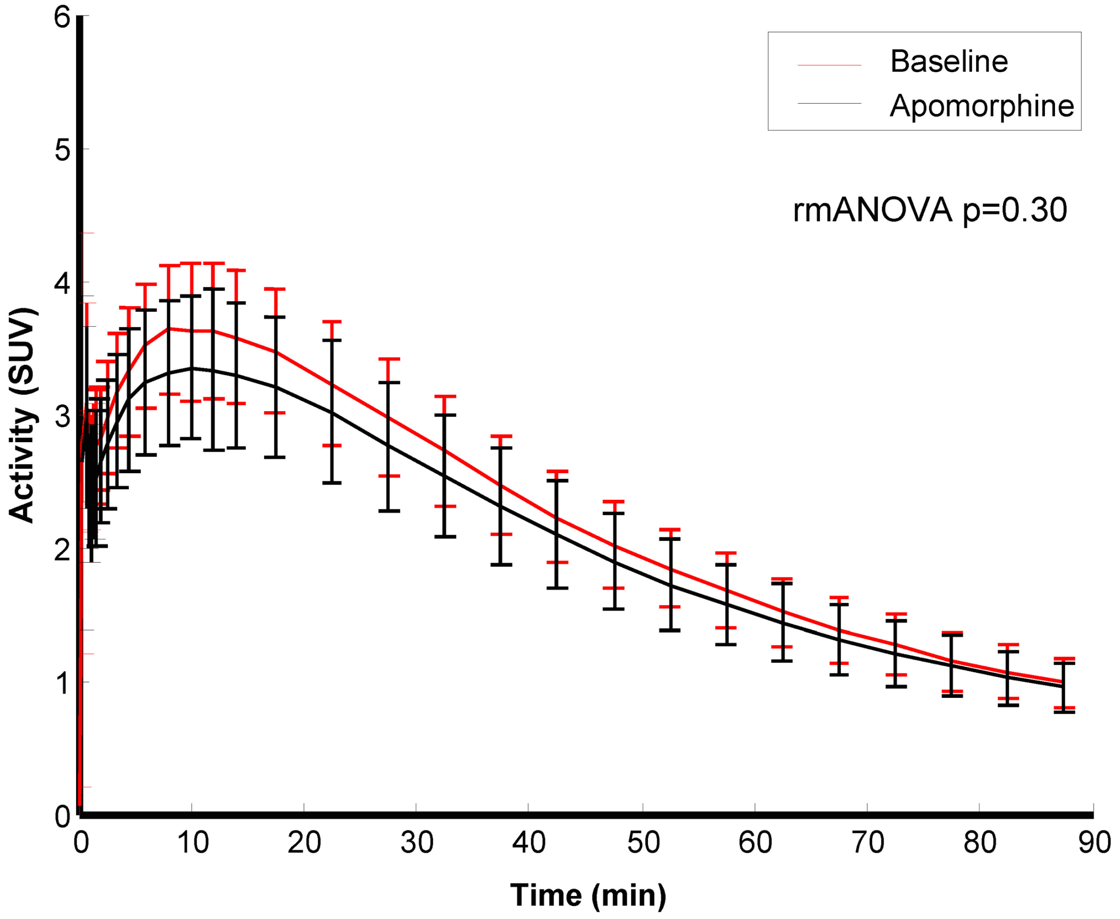

Supplement: Supplementary Material [file tp2016270x2.pdf]
